# Fatal Events in Cancer Patients Receiving Anticoagulant Therapy for Venous Thromboembolism

**DOI:** 10.1097/MD.0000000000001235

**Published:** 2015-08-14

**Authors:** Dominique Farge, Javier Trujillo-Santos, Philippe Debourdeau, Alessandra Bura-Riviere, Eva Maria Rodriguez-Beltrán, Jose Antonio Nieto, Maria Luisa Peris, David Zeltser, Lucia Mazzolai, Adrian Hij, Manuel Monreal, RIETE Investigators.

**Affiliations:** From the Assistance Publique-Hôpitaux de Paris, Saint-Louis Hospital, Internal Medicine and Vascular Disease Unit and Groupe Francophone on Thrombosis and Cancer, Paris7 Diderot University, Sorbonne Paris Cité, Paris, France (DF); Department of Internal Medicine, Hospital Universitario Santa-Lucía, Cartagena, Murcia, Spain (JT-S); Department of Oncology, Clinique Sainte Catherine, Groupe Francophone on Thrombosis and Cancer, Avignon, France (PD); Department of Vascular Medicine, Hôpital de Rangueil, Toulouse, France (AB-R); Department of Internal Medicine, Hospital Ntra, Sra. de Sonsoles, Avila, Spain (EMR); Department of Internal Medicine, Hospital General Virgen de la Luz, Cuenca, Spain (JAN); Consorcio Hospitalario Provincial de Castellón, Castellón, Spain (MLP); Department of Internal Medicine, Tel Aviv Soursky Medical Center, Tel Aviv, Israel (DZ); Department of Angiology, Centre Hospitalier Universitaire Vaudois, Lausanne, Switzerland (LM); Assistance Publique-Hôpitaux de Paris, Saint-Louis Hospital, Internal Medicine and Vascular Disease Unit and Groupe Francophone on Thrombosis and Cancer, Paris, France (AH); and Servicio de Medicina Interna, Hospital Universitari Germans Trias i Pujol, Badalona, Universidad Católica de Murcia, Spain (MM).

## Abstract

In cancer patients treated for venous thromboembolism (VTE), including deep-vein thrombosis (DVT) and pulmonary embolism (PE), analyzing mortality associated with recurrent VTE or major bleeding is needed to determine the optimal duration of anticoagulation.

This was a cohort study using the Registro Informatizado de Enfermedad TromboEmbólica (RIETE) Registry database to compare rates of fatal recurrent PE and fatal bleeding in cancer patients receiving anticoagulation for VTE.

As of January 2013, 44,794 patients were enrolled in RIETE, of whom 7911 (18%) had active cancer. During the course of anticoagulant therapy (mean, 181 ± 210 days), 178 cancer patients (4.3%) developed recurrent PE (5.5 per 100 patient-years; 95% CI: 4.8–6.4), 194 (4.7%) had recurrent DVT (6.2 per 100 patient-years; 95% confidence interval [CI]: 5.3–7.1), and 367 (8.9%) bled (11.3 per 100 patient-years; 95% CI: 10.2–12.5). Of 4125 patients initially presenting with PE, 43 (1.0%) died of recurrent PE and 45 (1.1%) of bleeding; of 3786 patients with DVT, 19 (0.5%) died of PE, and 55 (1.3%) of bleeding. During the first 3 months of anticoagulation, there were 59 (1.4%) fatal PE recurrences and 77 (1.9%) fatal bleeds. Beyond the third month, there were 3 fatal PE recurrences and 23 fatal bleeds.

In RIETE cancer patients, the rate of fatal recurrent PE or fatal bleeding was much higher within the first 3 months of anticoagulation therapy.

## INTRODUCTION

Despite appropriate anticoagulation, cancer patients with acute venous thromboembolism (VTE), defined as deep-vein thrombosis (DVT) or pulmonary embolism (PE), are at increased risk for VTE recurrences and major bleeding compared to noncancer patients.^1–5^ Based on randomized clinical trials^6–8^ and meta-analyses,^9^ specific guidelines recommend that cancer patients with VTE receive initial therapy with low-molecular-weight heparin (LMWH), Fondaparinux or unfractionated heparin (UFH), followed by “early maintenance” (10 days to 3 months) and “long-term treatment” (beyond 3 months) with LMWH, rather than with vitamin K antagonists (VKA).^10–14^ Use of LMWH from 10 days up to 3 months has been established by 3 randomized clinical trials^6–8^ with a high level of evidence, but there is no consensus regarding the optimal duration or the intensity of anticoagulation beyond 3 months. It is generally accepted that cancer patients should receive LMWH during at least 3 months for established VTE up to 6 months since in the largest study in this setting cancer patients were treated for 6 months.^7^ In the absence of data, the decision regarding the termination or continuation of anticoagulation beyond the first 3 or 6 months is largely based on individual evaluation of the benefit-risk ratio, while considering the risk of VTE recurrences against the risk of major bleeding, tolerability, patients’ preference and cancer activity.^12,13^

The mortality associated with recurrent VTE and major bleeding provides useful information to balance the respective risks and benefits of anticoagulation, but most clinical trials were underpowered to assess the respective risk fatal VTE or fatal bleeding.^15^ Furthermore, a number of patients are often excluded from randomized trials of anticoagulant therapy because of comorbid conditions, disseminated cancer, short-life expectancy, or contraindications, and therefore anticoagulation regimens based on the results from randomized clinical trials might not be applicable to all patients with active cancer and VTE.

The *R*egistro *I*nformatizado de *E*nfermedad *T*rombo*E*mbólica (RIETE) Registry is an ongoing, international, multicenter, prospective registry of consecutive patients presenting with symptomatic, acute VTE. Its methodology has been described previously.^16–18^ In the current analysis, we assessed the influence of initial VTE presentation—defined as DVT (including those on central venous catheter) or PE—on the mortality rate due to VTE recurrences and major bleeding throughout the whole duration of anticoagulant treatment, which included use of LMWH, UFH, Fondaparinux, or VKA. We also compared the rate of fatal recurrent PE and fatal bleeding over time under the various types of anticoagulation throughout follow-up.

## METHODS

Consecutive patients with symptomatic acute VTE confirmed by objective tests (contrast venography or ultrasonography for suspected DVT, pulmonary angiography, lung scintigraphy, or helical computed tomography scan for suspected PE) were enrolled in RIETE. Patients were excluded if currently participating in a therapeutic clinical trial with a blinded therapy. All patients provided written or oral consent for participation in the registry, in accordance with local ethics committee requirements. Participating physicians ensured that eligible patients were consecutively enrolled. Data recorded on a computer-based case report form at each participating hospital were submitted to a centralized coordinating center through a secure website. Data quality was regularly monitored as previously described.^16–18^

### Study Design and Outcomes

For this analysis, cancer patients with newly diagnosed cancer (less than 3 months earlier) or with cancer being treated by either surgery, chemotherapy, radiotherapy, hormonal, support therapy, or combined treatments, and receiving anticoagulation (LMWH, UFH, thrombolytics, inferior vena cava filter (VCF), VKA, or Fondaparinux) for acute VTE were considered. VTE initial presentation, clinical characteristics, cancer site and staging, treatment options and outcome during the course of anticoagulation were compared. Major outcomes were fatal (recurrent) PE and fatal bleeding. Fatal PE, in the absence of autopsy, was defined as any death appearing within the first 10 days after PE diagnosis (either the initial PE episode or recurrent PE), in the absence of any alternative cause of death. Fatal bleeding was defined as any death occurring within 10 days of a major bleeding episode, in the absence of an alternative cause of death. Secondary outcomes were the development of DVT or PE recurrences, major bleeding events and all-cause of death.^18^

Bleeding complications were classified as “major” if they were overt and required a transfusion of 2 units of blood or more, or were retroperitoneal, spinal or intracranial, or when they were fatal.^19^ Most outcomes were classified as reported by the clinical centers. However, if staff at the coordinating center were uncertain how to classify a reported outcome, that event was reviewed by a central adjudicating committee (less than 10% of events).

### Baseline Variables

The following parameters were recorded when the qualifying episode of VTE was diagnosed: patient's gender, age, and body weight; presence of coexisting conditions (chronic heart or lung disease) and additional VTE risk factors including recent immobilization (defined as nonsurgical patients confined to bed with bathroom privileges for ≥4 days in the 2 months before VTE diagnosis), and surgery (defined as an operation in the 2 months before VTE); recent (<30 days before VTE) major bleeding; clinical characteristics of the malignancy (cancer site, staging, cancer duration since diagnosis); and laboratory data on admission, including serum creatinine levels.

### Treatment and Follow-up

Patients were managed according to the local clinical practice of each participating hospital, that is, there was no standardization of anticoagulation treatment. The type (LMWH, UFH, thrombolytics, VCF, VKA, or Fondaparinux), dose of LMWH and duration for each type of anticoagulation were recorded up to 10 days (initial treatment), between 10 days and 3 months (early maintenance), and after 3 months (long-term therapy). During each visit after onset of VTE, either in or out the hospital after discharge or in the outpatient clinic, any signs or symptoms suggesting either VTE recurrences or bleeding complications were noted. Clinically suspected recurrent DVT or PE was investigated by repeated imaging as appropriate according to local physician practice.

### Statistical Analysis

Categorical variables were reported as percentages and compared using the Chi-square test (2-sided) and Fisher exact test as appropriate. Odds ratios and corresponding 95% confidence intervals (CIs) were calculated, and a *P*-value <0.05 was considered to be statistically significant. Continuous variables were compared with a Student *t* test. Incidence rates were calculated as cumulative incidence (events/100 patient-years). The case-fatality rate (CFR) of recurrent PE and major bleeding, defined as the proportion of patients who die as a consequence of these conditions, was calculated. Statistical analyses were conducted with SPSS software for Windows release 15.0 (SPSS Inc., Chicago, Illinois, USA).

## RESULTS

From March 2001 up to January 2013, 7911 (18%) out of the 44,794 patients enrolled in RIETE had active cancer. Of these, 4125 cancer patients initially presented with PE (with or without concomitant DVT) and 3786 with DVT alone. Their clinical characteristics are depicted in Table 1. Mean age and body weight were higher in cancer patients presenting with PE than in those with DVT alone, and they more likely had additional risk factors for VTE, including immobilization >4 days, postoperative status or renal insufficiency, whereas those with DVT alone had had more frequent previous VTE. Lung cancer was more frequent in patients treated for initial PE, and uterine cancer than in those treated for DVT alone. Most cancer patients (92%) were initially treated with LMWH, but a higher proportion of those presenting with PE as compared to those presenting with DVT alone received UFH (Table 2). Thereafter, 58% of cancer patients continued receiving LMWH at least during the first 3 months and 35% switched to VKA. LMWH was initially preferred over VKA in patients with disseminated cancer (62% received LMWH, 33% VKA) or anemia (66% vs. 30%, respectively). The overall duration of anticoagulation was longer in cancer patients initially presenting with PE than in those with DVT alone (191 ± 242 days vs. 171 ± 201 days), but mean daily LMWH doses were similar.

**TABLE 1:**
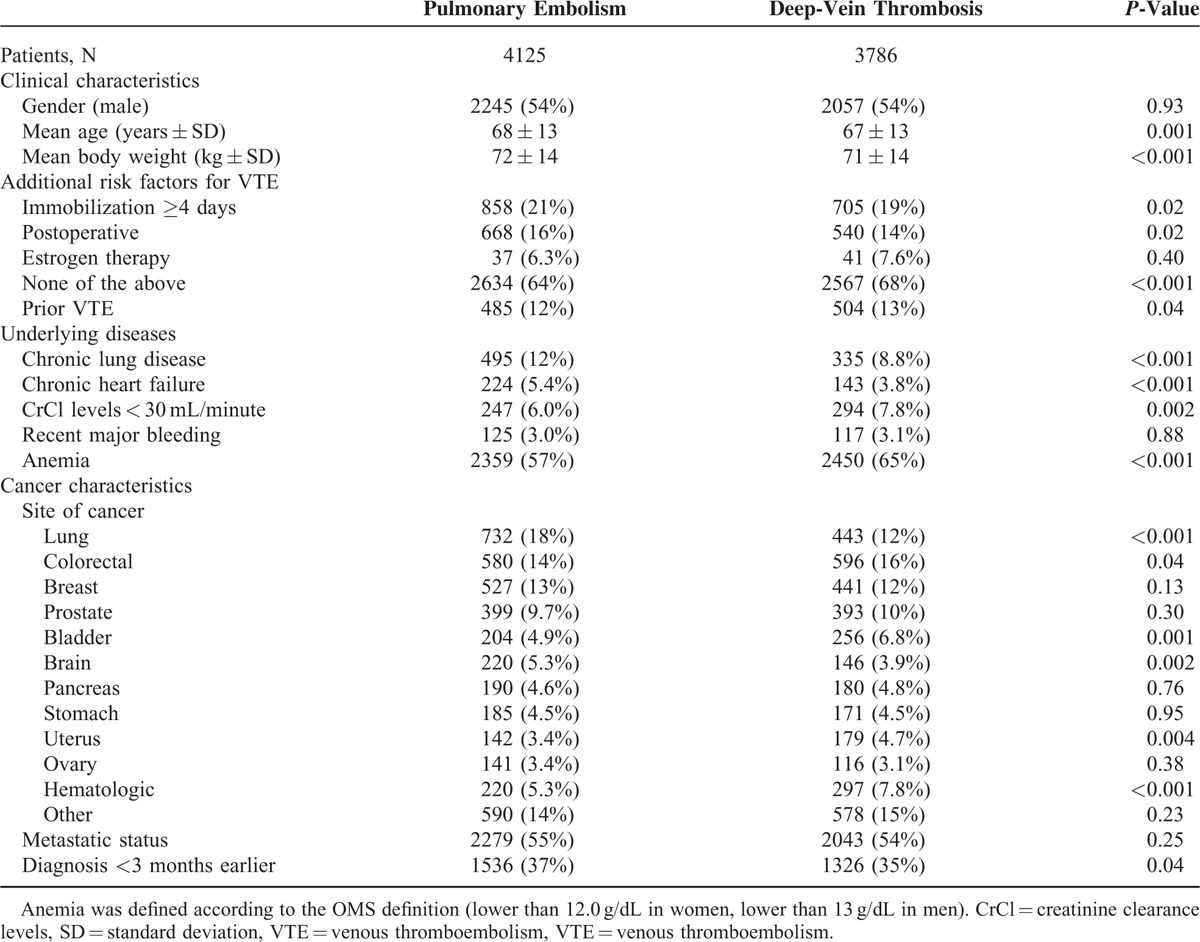
Clinical Characteristics and Additional Risk Factors for Venous Thromboembolism (VTE) According to Initial VTE Presentation in 7911 Cancer Patients Enrolled in RIETE and Treated for VTE

**TABLE 2:**
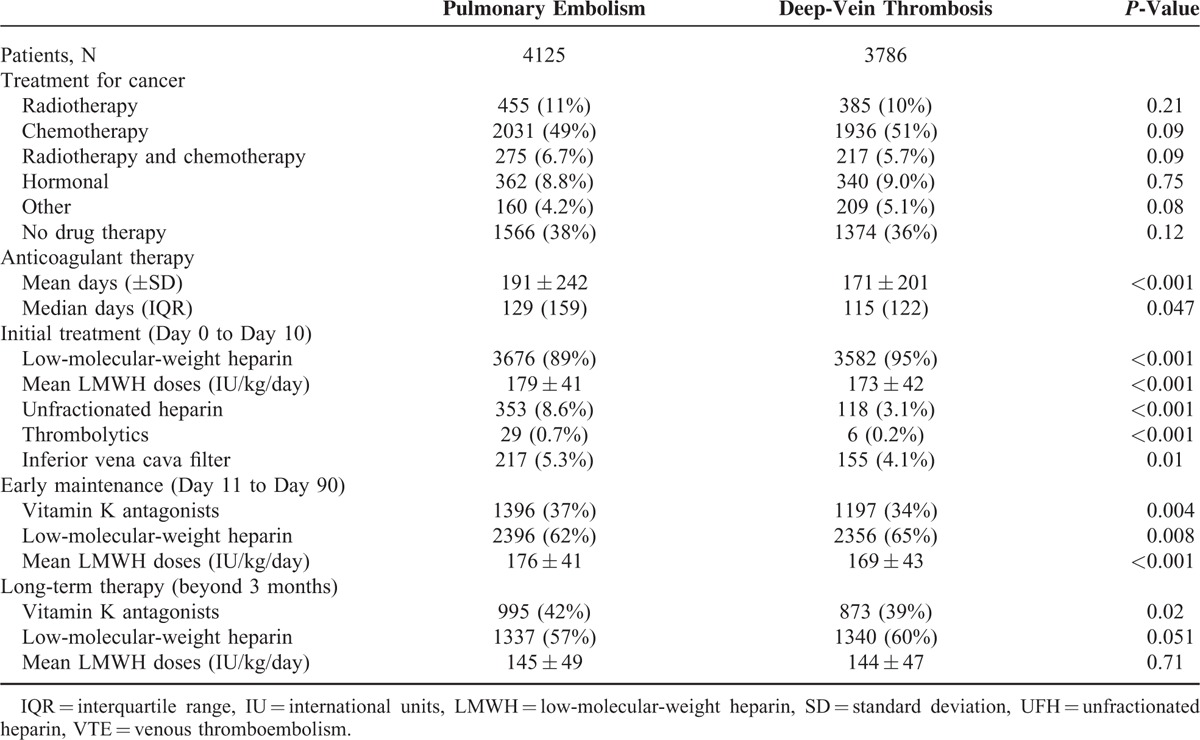
Treatment Strategies According to Initial VTE Presentation

### PE Recurrences

**FIGURE 1:**
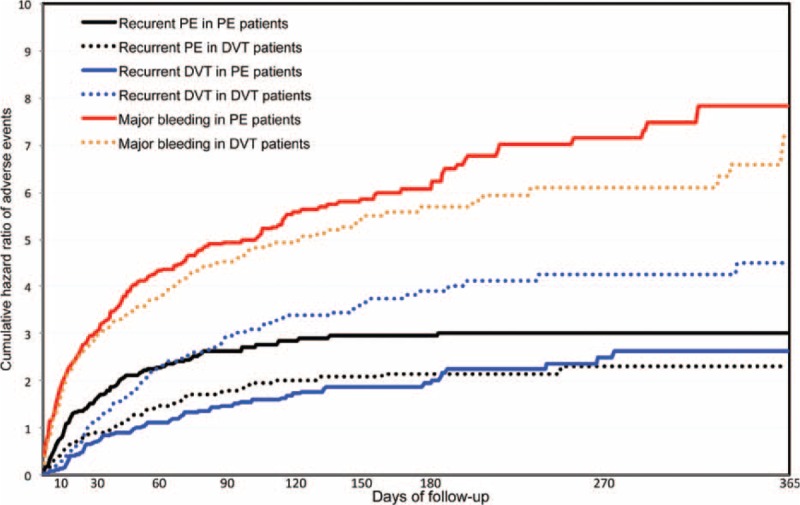
Cumulative rate of recurrent pulmonary embolism (PE), recurrent deep-vein thrombosis (DVT) and major bleeding within the first 12 months of anticoagulation, according to initial venous thromboembolism (VTE) presentation (PE, with or without DVT, or DVT alone) in 7911 cancer patients enrolled in RIETE and treated for VTE.

During the course of anticoagulation, 178 cancer patients developed PE recurrences (5.5 per 100 patient-years; 95% CI: 4.8–6.4). Of these, 51 occurred within the first 10 days of anticoagulation (40 under LMWH, 5 under VKA, 6 other), 105 from Days 11 to 90 (78 on LMWH, 22 VKA, 5 other) and 22 after Day 91 (14 on LMWH, 8 VKA) (Figure 1). PE recurrences most likely occurred in patients initially presenting with PE than in those with DVT alone (6.4 vs. 4.6 per 100 patient-years; *P* = 0.03) (Table 3). Forty-eight patients (27%) died within less than 24 hours after recurrent PE, with minimal time to change therapy; 62 continued with the same therapy (same drug and doses), 47 received higher doses, 21 moved to LMWH, and VCF was placed in 18. Seventy-three patients (41%) died within the first 2 weeks, of whom 62 (85%) died of the recurrent PE event. Overall, PE recurrences developed more likely in patients receiving long-term LMWH than in those on AVK, but this may be due to the higher proportion of patients with metastatic cancer receiving LMWH than AVK (Table 4).

**TABLE 3:**
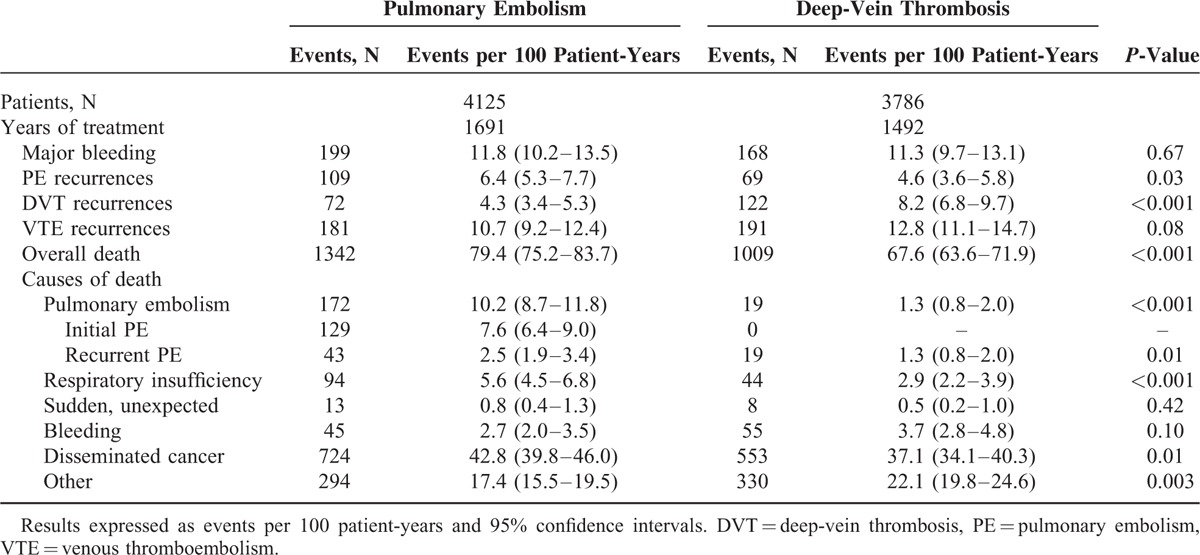
Clinical Outcome During the Course of Anticoagulant Therapy in 7911 Cancer Patients Reported to RIETE According to Initial VTE Presentation

**TABLE 4:**
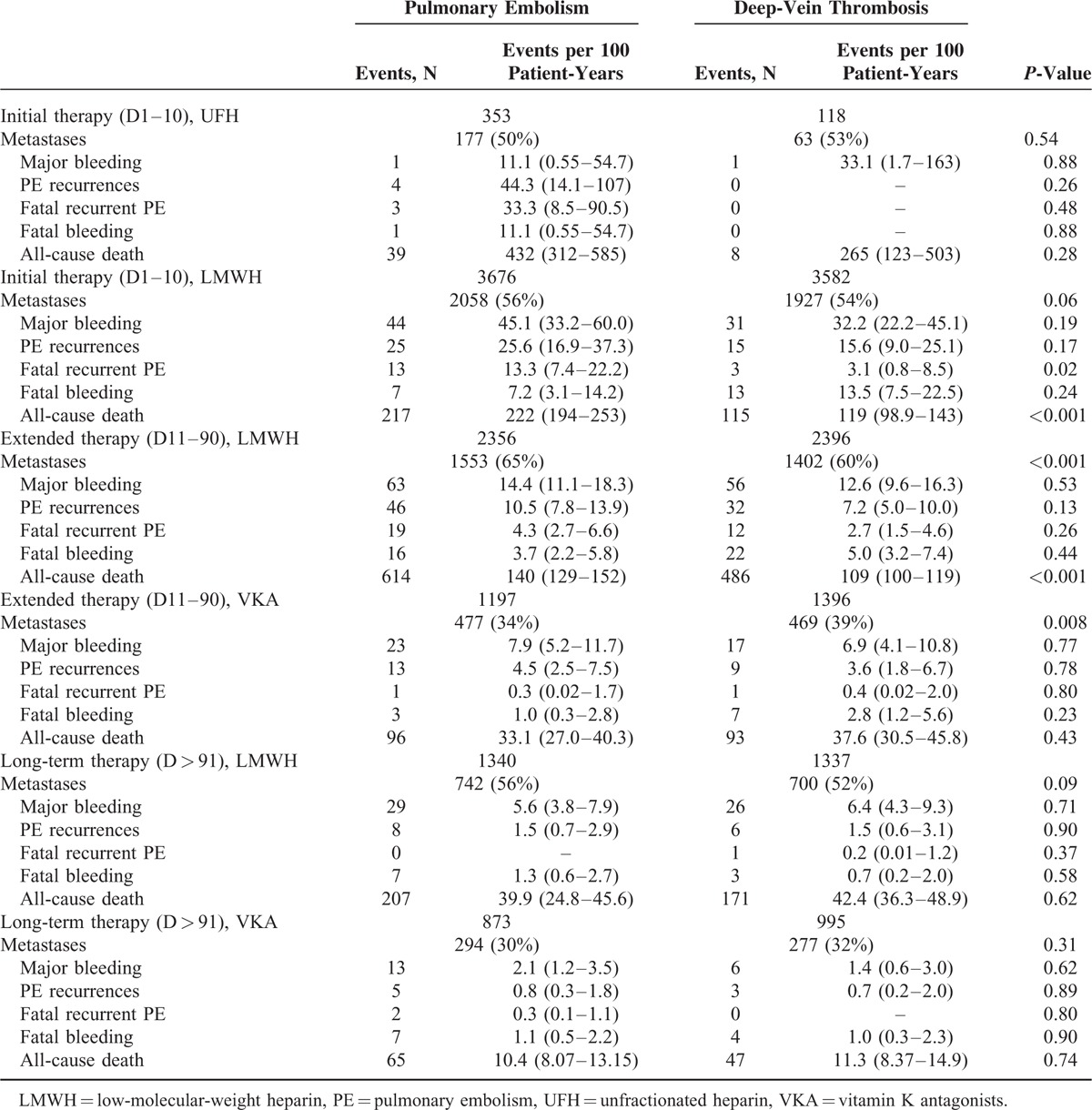
Clinical Outcome During the Course of Anticoagulation, According to Initial VTE Presentation, Period of Therapy and Prescribed Drugs

### DVT Recurrences

During the course of anticoagulation, 194 patients presented with recurrent DVT (6.2; 95% CI: 5.3–7.1). Of these, 17 recurrences appeared within the first 10 days of therapy (13 on LMWH, 3 VKA, 1 UFH), 130 during “early maintenance” (76 on LMWH, 54 VKA) and 47 during “long-term” treatment beyond 3 months (28 on LMWH, 19 VKA). DVT recurrences were more likely to appear in cancer patients initially presenting with DVT (8.2 vs. 4.3 per 100 patient-years; *P* < 0.001) than in those with PE. The type and dose of anticoagulation treatment was not modified in 105 cancer patients treated for VTE recurrence, 38 received higher doses of whom 2 died of bleeding after increasing heparin doses, 49 were moved to LMWH, and a VCF was placed in 8 patients.

### Major Bleeding

**FIGURE 2:**
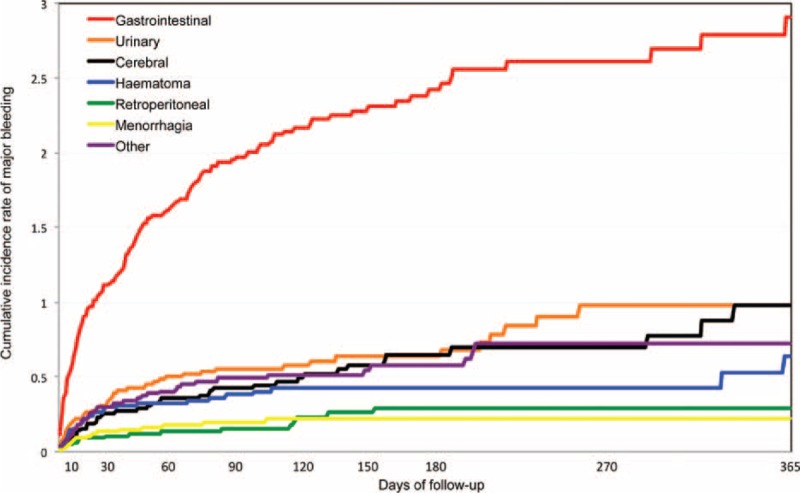
Cumulative incidence of major bleeding (and sites of bleeding) within the first 12 months of anticoagulation in 7911 cancer patients enrolled in RIETE and treated for venous thromboembolism (VTE) (pulmonary embolism (PE), with or without deep-vein thrombosis (DVT), or DVT alone).

During the overall duration of anticoagulation, 367 cancer patients presented major bleeding (11.3 per 100 patient-years; 95% CI: 10.2–12.5). Of these, 249 (68%) were receiving LMWH, 109 (30%) VKA and 9 (2.5%) were on other treatments including UFH. Anticoagulation was discontinued in 237 patients (65%) (for <5 days in 172, ≥5 days in 65), 46 (13%) moved to low-dose LMWH and a VCF was placed in 36 (9.8%). Major bleeding occurred within the first 10 days of therapy in 126 cancer patients (75 under LMWH, 48 VKA, 3 other), in 165 during “early maintenance” (119 on LMWH, 40 VKA, 6 other), and in 76 during “long-term” treatment (55 on LMWH, 21 VKA). The rate of major bleeding was similar in patients initially presenting with initial PE or DVT alone (Table 3). During the first 3 months of anticoagulation, the most common sites of major bleeding were the gastrointestinal (GI) tract (n = 137), urinary (n = 39), brain (n = 29), hematoma (n = 27), menorrhagia (n = 14), and retroperitoneal (n = 11) (Figure 2). After the third month, the most common sites of major bleeding were the GI tract (n = 33), brain (n = 16), urinary (n = 7), and retroperitoneal (n = 5). Sixty-five (18%) of cancer patients with major bleeding died in <24 hours, 130 (35%) within the first 2 weeks. Of these, 104 (80%) died of bleeding and 4 (3.1%) died of recurrent PE (shortly after discontinuing anticoagulation). Overall, major bleeding appeared more likely in patients receiving long-term LMWH than in those on AVK, but this may be due to the higher proportion of patients with metastatic cancer receiving LMWH than AVK (Table 4).

### Mortality

**FIGURE 3:**
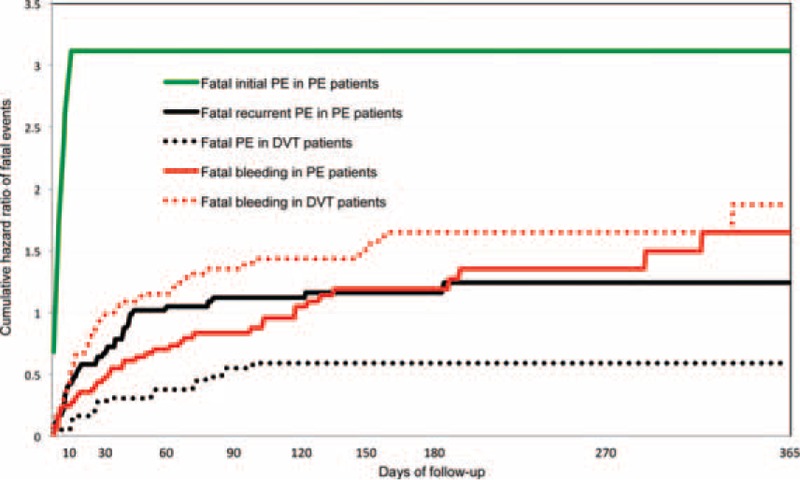
Cumulative rate of fatal pulmonary embolism (PE) and fatal bleeding within the first 12 months of anticoagulation, according to initial venous thromboembolism (VTE) presentation, PE with or without deep-vein thrombosis (DVT), or DVT alone in 7911 cancer patients enrolled in RIETE and treated by anticoagulation.

During anticoagulation for VTE, 2351 cancer patients died (71.5 per 100 patient-years; 95% CI: 68.6–74.5). The most common causes of death were: disseminated malignancy (n = 1277), PE (n = 191), respiratory insufficiency (n = 138, of whom 48 had lung cancer, 32 chronic lung disease, and 17 chronic heart failure), and bleeding (n = 100). Of 191 cancer patients with fatal PE, 129 died of the initial PE and 62 of recurrent PE. Among 100 cancer patients with fatal bleeding, the most common sites of bleeding were the GI tract (n = 51), and the brain (n = 19). During the first 3 months of anticoagulation, 59 fatal PE recurrences and 77 fatal bleeds occurred (Figure 3). Beyond the third month, there were 3 fatal PE recurrences and 23 fatal bleeds. During the first 3 months of anticoagulation, the CFR of recurrent PE was 37.8% (95% CI, 30.2–45.9), compared to 13.6% (95% CI, 3.0–44.9) beyond the third month. The CFR of major bleeding was 26.3% (95% CI, 21.4–31.7) during the first 3 months, and 31.1% (95% CI, 20.8–42.9) beyond this period.

One in every 4 cancer patients dying of recurrent PE (26%) or bleeding (27%) had no metastases, 1 in every 2 (61% and 34%, respectively) were aged <65 years, and 1 in every 10 patients dying of bleeding had a history of recent bleeding before the index VTE (Table 5). Moreover, patients with prostate, bladder, stomach, or uterine cancers less likely died of PE than of bleeding, while those with breast cancer more likely died of PE than of bleeding. Finally, 21 patients had a sudden, unexpected death. Unfortunately, no necropsy studies were performed, and there is no way to know how many of them (if any) died of PE or bleeding.

**TABLE 5:**
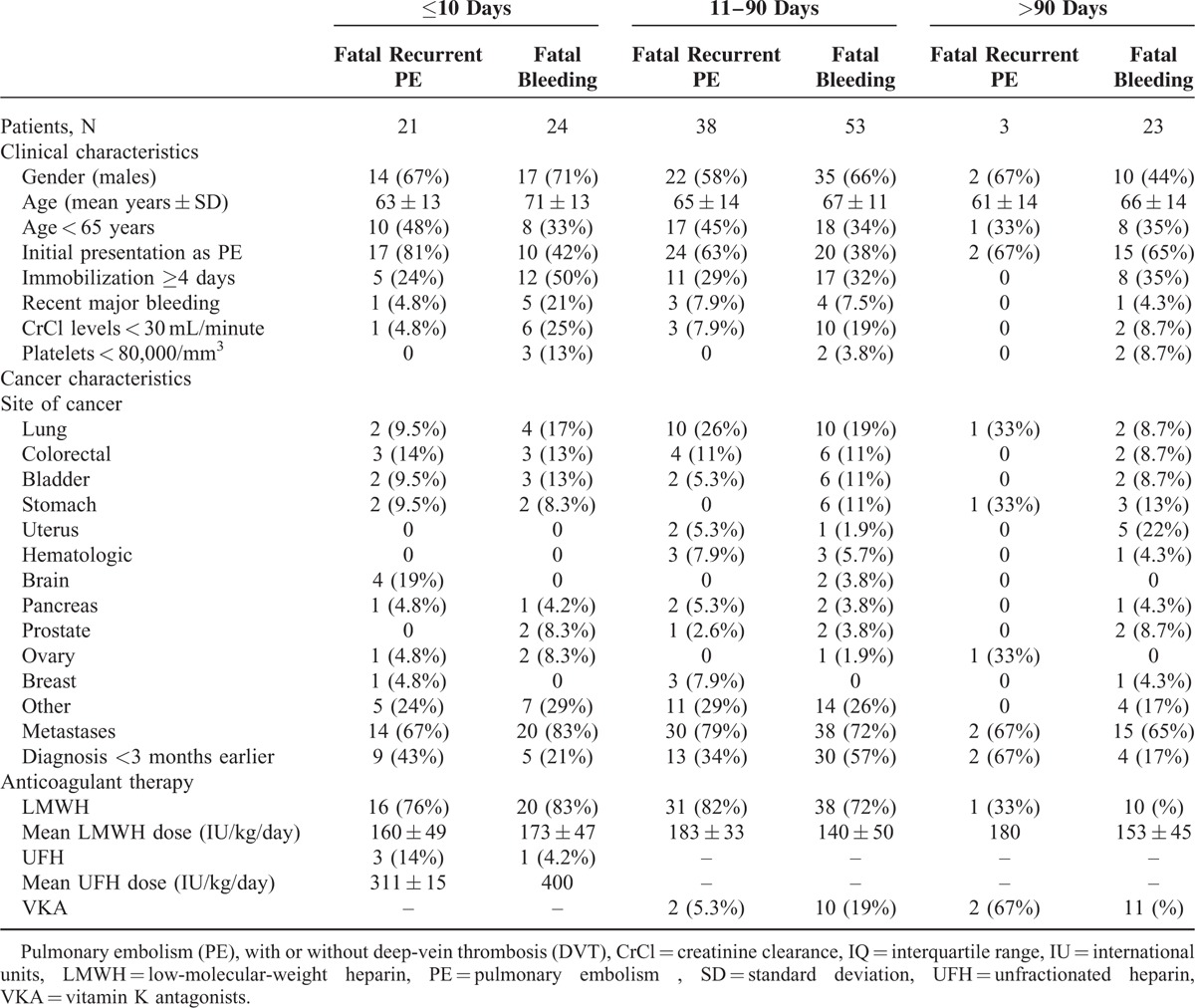
Clinical Characteristics and Treatment in Cancer Patients Enrolled in RIETE and Treated by Anticoagulation for VTE With Fatal Recurrent PE or Fatal Bleeding According to Time of Presentation

## DISCUSSION

This prospective multicenter RIETE cohort analysis of cancer patients treated for acute VTE, obtained from a large number of consecutive cancer patients treated for VTE, allowed to analyze a large cohort of cancer patients treated by anticoagulation at best according to these guidelines or according to local practice. In this specific cancer population treated for VTE, the overall rate of VTE recurrences during anticoagulation was close to the rate of major bleeding (372 events vs. 367 events), but mortality due to recurrent PE was lower than the mortality due to bleeding (62 vs. 100 deaths, respectively), particularly after the third month of anticoagulation (3 vs. 23 deaths, respectively).

These results confirm 2 recent studies showing that the CFR of recurrent VTE and major bleeding were similar during initial treatment of VTE in cancer patients, but that the CFR of recurrent VTE decreased after the third month of anticoagulation.^15,20^ The higher risk of dying from bleeding than from PE after the first 3 months of anticoagulation suggests that a less aggressive anticoagulant strategy (or a shorter duration) might reduce fatal bleeding, but could also in turn increase the incidence of PE.

We also found that, irrespective of the type of anticoagulant drugs, the risk of dying from recurrence of PE during anticoagulation treatment, varied according to VTE presentation at baseline. In patients initially presenting with PE, the risk of fatal recurrent PE during anticoagulation and the risk of fatal bleeding were similar. The lower risk of dying from recurrent PE in patients initially presenting with DVT can be attributed to the fact that DVT patients most likely recurred as DVT, and no patient with recurrent DVT died of PE. The dose of anticoagulation treatment did not appear to account for difference in outcomes between cancer patients presenting with VTE alone or with PE, and the rate of fatal bleeding was similar over time. The much lower risk of dying from PE than from bleeding in patients with DVT has been previously reported also in the elderly (with or without cancer),^21^ and in patients with renal insufficiency.^22^ Patients with prostate, bladder, stomach, or uterine cancers were less likely to die from PE than from bleeding and patients with breast cancer died more of PE than of bleeding, underlying the need for tailored anticoagulation regimen according to the cancer type in the future.

The incidence of 11 VTE recurrences per 100 patient-years observed in our study is similar to results from the control arms of randomized clinical trials using VKA after 10 days as compared to long-term LMWH for 3^6,8^ or 6 months,^7^ but the incidence of 11 major bleeding events per 100 patient-years was over 2-fold higher than reported in these trials.^6–8^ The higher incidence of major bleeding in the current analysis reflects enrollment of consecutive unselected patients, including more patients with metastatic cancer and patients with multiple risk factors for bleeding who are often excluded from randomized trials. The RIETE registry provides data on the treatment and outcome of VTE in a real-world situation with an unselected patient population, in contrast to the rigorously controlled conditions of randomized clinical studies. There was a relatively low rate of VCF insertion, although 10% of the patients dying of bleeding had a history of recent bleeding before the index VTE. A recent RIETE study in 371 patients (of whom 60 with cancer) has shown that inferior VCF insertion, as compared with anticoagulant therapy, in patients with acute symptomatic VTE and a significant risk of bleeding was associated with a lower risk of PE-related death, but did not decrease the rate of major bleeding at 30 days.^23^

In the literature, no studies have yet assessed the efficacy and safety of anticoagulant therapy beyond the first 6 months, but in a recent survey containing 49 questions on different aspects of the treatment of cancer patients with VTE, almost half of the respondents chose to continue LMWH treatment after the initial 3 to 12 months, most often for life-long.^24^ According to our findings, indefinite anticoagulation should probably be reconsidered for some of these patients.

The present study has a number of potential limitations. First, patients were not treated with a standardized anticoagulant regimen and treatment varied with local practice, which was influenced by a physician's assessment of a patient's risk of bleeding. Second, data from registries are susceptible to selection bias if a nonrepresentative sample of patients is selected for analysis. However, the RIETE registry captured a broad range of cancer patients with acute symptomatic VTE from multiple medical centers, countries, and treatment settings, and the study cohort was less likely a skewed population. Third, to fulfill the definition of fatal PE in RIETE patients must first experience an objectively confirmed recurrent PE followed by death within 10 days. Thus, all sudden unexplained deaths, usually considered as “likely” fatal recurrent PE, and many patients dying of respiratory insufficiency are not considered in this analysis. Therefore, the rate of fatal PE may have been underestimated, especially after hospital discharge. However, over 50% of these patients had chronic lung or heart disease, or lung cancer, and objective tests ruled out PE in some of them. Moreover, some deaths occurring at home without diagnosis may also have been due to cerebral bleeding. Finally, the study did not use a central committee to assign cause of death, but the number of deaths in the registry renders this task virtually impossible. On the other hand, strengths of the current analysis include that a large number of consecutive unselected patients were enrolled, and that fatal PE and fatal bleeding are by far the most important outcomes during the treatment of acute DVT. When considering the trade-off between increases in bleeding and decreases in recurrent VTE with different approaches to management, fatal PE and fatal bleeding are of equal importance, whereas that is not the case when nonfatal episodes of recurrent VTE and bleeding are included (eg, a recurrent DVT and a nonfatal intracranial bleed).

In conclusion, this large prospective RIETE study showed that the risk of dying from recurrent PE, varied according to VTE presentation at baseline, irrespective of the type of anticoagulation, and also according to cancer characteristics. In patients initially presenting with DVT, the risk of dying from recurrent PE was lower than in those initially presenting with PE. The overall rate of fatal recurrent PE was close to the rate of fatal bleeding, but was much higher within the first 3 months of anticoagulation therapy, suggesting that a less aggressive anticoagulant strategy (or a shorter duration) might reduce fatal bleeding over time. Implementation of current guidelines in cancer patients with VTE would benefit from randomized trials, which are needed to establish whether any different strategy is superior to standard anticoagulation after 3 months in cancer patients with VTE.

## APPENDIX

*Members of the RIETE Group*: SPAIN: Alcalde M, Arcelus JI, Ballaz A, Barba R, Barrón M, Barrón-Andrés B, Bascuñana J, Bedate P, Blanco-Molina A, Bueso T, Casado I, Conget F, del Molino F, del Toro J, Falgá C, Fernández-Capitán C, Fuentes MI, Gallego P, García J, García-Bragado F, Gavín O, Gómez V, González J, González-Bachs E, Grau E, Guil M, Guijarro R, Gutiérrez J, Hernández L, Jara-Palomares L, Jaras MJ, Jiménez D, Jiménez S, Lobo JL, López-Jiménez L, López-Sáez JB, Lorente MA, Lorenzo A, Luque JM, Madridano O, Macià M, Maestre A, Marchena PJ, Martín M, Monreal M, Mora JM, Muñoz FJ, Nauffal MD, Nieto JA, Núñez MJ, Ogea JL, Otero R, Pedrajas JM, Peris ML, Riera-Mestre A, Rivas A, Rodríguez-Dávila MA, Román P, Rosa V, Ruiz J, Ruiz-Ribó MD, Ruiz-Gamietea A, Ruiz-Giménez N, Sahuquillo JC, Samperiz A, Sánchez Muñoz-Torrero JF, Soler S, Tiberio G, Tilvan RM, Tolosa C, Trujillo J, Uresandi F, Valdés M, Valero B, Valle R, Vela J, Vidal G, Villalobos A, Villalta J, BRAZIL: Gadelha T, CZECH REPUBLIC: Malý R, Hirmerova J, Tomko T. FRANCE: Bertoletti L, Bura-Riviere A, Farge-Bancel D, Grange C, Hij A, Mahe I, Merah A, Quere I. GERMANY: Schellong S, GREECE: Babalis D, Papadakis M, Tzinieris I, IRELAND: Faul J, ISRAEL: Braester A, Brenner B, Tzoran I, Zeltser D, ITALY: Barillari G, Ciammaichella M, Dalla Valle F, Di Micco P, Duce R, Maida R, Pasca S, Piovella C, Poggio R, Prandoni P, Quintavalla R, Rocci A, Rota L, Schenone A, Tiraferri E, Tonello D, Tufano A, Visonà A, Zalunardo B, PORTUGAL: Brinquinho M, Gomes D, Gonçalves F, Santos M, Saraiva M, REPUBLIC OF MACEDONIA: Bosevski M, Kovacevic D, SWITZERLAND: Alatri A, Aujeski D, Bounameaux H, Calanca L, Mazzolai L, UNITED STATES: Caprini J.
